# Effects of Psychological Discomfort on Social Networking Site (SNS) Usage Intensity During COVID-19

**DOI:** 10.3389/fpsyg.2022.939726

**Published:** 2022-07-22

**Authors:** Hyeon Jo

**Affiliations:** Department of Strategic Planning, RealSecu, Busan, South Korea

**Keywords:** COVID-19, social networking site, SNS, social distancing, risk perception, cabin fever syndrome, information technology

## Abstract

To cope with the COVID-19 pandemic, many countries are implementing social measures. Social distancing, working from home, and non-face-to-face lectures have led to major changes in people's activities. Since face-to-face classes are restricted, students in higher education become to feel psychological and cognitive discomforts such as isolation and risk perception. The purpose of this study is to explore the effects of psychological discomforts on the social network site (SNS) usage intensity of University students. Using structural equation modeling (SEM), this study applied SmartPLS 3.3.9 to analyze 271 valid samples. The results show that the affective risk perception significantly affects social distancing attitude. Cognitive risk perception is positively related to social distancing intention. In addition, cabin fever syndrome influences SNS usage intensity, affective risk perception, and cognitive risk perception. In conclusion, psychological discomfort partially affects the intensity of SNS use. Therefore, the government should set policies by reflecting citizens' mental difficulties and SNS activities together. Moreover, companies are needed to carefully consider the risk of the sense of isolation when marketing to SNS users.

## Introduction

Since the COVID-19 outbreak, governments around the world have suggested that people stay at home and reduce their outdoor activities (Yarimkaya and Esentürk, 2020). Citizens are participating in preventive measures such as social distancing, mask wearing, working from home, and non-face-to-face learning. As social distancing and lockdown continue, people have been interacting with the outside world by using social network sites (SNSs) more actively (Nabity-Grover et al., 2020). According to statistics, the global usage of SNS, including Facebook, Instagram, and WhatsApp, has drastically increased (Holmes, 2020; Vall-Roqué et al., 2021). SNS has been found to facilitate the healthy behavior of residents, which in turn reduce negative mood and social distancing aroused from social isolation (Qin et al., 2020). In particular, SNS plays a vital role in young individuals' lives (Gioia et al., 2020; Vall-Roqué et al., 2021). Therefore, it would be meaningful to pay attention to the SNS activities of University students, a young group whose external activities have been restricted by social measures.

Social distancing helps to decrease pressure on health services and hampers the spread of COVID-19 (Koo et al., 2020). It is the most effective way to block the virus and prevent infection (Adiyoso and Wilopo, 2020). People make decisions based on social distancing steps noticed on media. The greater the attitude and intention toward social distancing, the fewer outdoor activities, which would affect the use of SNS. Thus, this study posits that social distancing attitude and social distancing intention are vital components that lead to SNS usage intensity.

The emergence of COVID-19 and corresponding social distancing have created derivative psychological discomfort, respectively. First, COVID-19 itself makes people aware of risks such as infection and death (Dryhurst et al., 2020). Traumatic stress and fear may be included in the psychological impact caused by COVID-19 (Chakraborty et al., 2020). Fear means worrying about getting affected by COVID-19 (Wang et al., 2020). Risk perception in the COVID-19 situation also measures anxiety about the infection or death (Ju and You, 2022). Psychological risk occurs with mental discomfort. It represents the effects of COVID-19 (Chua et al., 2021). Since psychological impact generally involves mental discomfort and the notion of risk perception under the COVID-19, this study considers risk perception as a component of psychological discomfort. Second, social distancing/lockdown limits citizens' outside activities and increases isolation time (Van Orden et al., 2021). It causes cabin fever syndrome (Estacio et al., 2020). Cabin fever refers to mental discomfort experienced in confined spaces for a long period (Crawford and Crawford, 2021). In particular, social lockdown gives children and young people the psychological stress of cabin fever (Crawford and Crawford, 2021). In summary, COVID-19 itself causes people mental discomfort of risk perception, and social measures cause cabin fever syndrome. Therefore, this research considers risk perception and cabin fever as factors of psychological discomfort.

As the development of COVID-19 proceeds globally, it is becoming increasingly significant to figure out public risk perception (Van Bavel et al., 2020). Risk perception is significant in determining health-protective behavior (Savadori and Lauriola, 2021). Higher perceived risk can elevate an individual's adherence to protective actions (Brewer et al., 2007). In this vein, people with a higher level of risk perception for COVID-19 might regard social distancing as more beneficial and form a greater degree of behavioral intention. Risk perception is measured based on two dimensions, affective and cognitive (Brug et al., 2004). Therefore, this study investigates the role of affective risk perception and cognitive risk perception in capturing SNS users' attitudes and intentions toward social distancing.

Cabin fever syndrome is described as a common reaction when people are confined in a space for a long time (Seitz, 2019). Hoof (2020) stated that COVID-19 lockdown might result in a secondary epidemic of mental stress and listlessness. In the context of the pandemic, quarantined individuals would experience cabin fever syndrome because of stress due to social distancing (Chakraborty et al., 2020). People with higher cabin fever syndrome may increase SNS usage to address the sense of isolation and closure. They also might have a higher perception of the COVID-19 risk. Hence, this study posits cabin fever syndrome as the predominant factor in forming SNS usage intensity, affective risk perception, and cognitive risk perception.

Some previous studies have analyzed the factors affecting the educational outcome of University students in the COVID-19 environment in various ways. Chen et al. (2022) demonstrated the antecedents of English language learning outcomes during COVID-19. The authors revealed that self-concept and self-efficiency are the vital factors influencing the results of University students. Iqbal et al. (2021) suggested the research model identifying the key determinants of cognitive outcomes of University students. They showed that self-awareness, empathy, motivation, and social skills had a significant effect on relational engagement. In addition, relational engagement was figured to be a significant leading variable of cognitive outcomes. At the same time, Iqbal et al. (2022) clarified the factors affecting the study habits of University students. They pointed out that study habits were influenced by self-awareness, self-motivation, regulation of emotions, and cognitive engagement.

The aforementioned studies did not reflect the psychological factors of University students due to the COVID-9. COVID-19 has changed the psychological state of the students, and it may have affected their social media activities. Therefore, this paper aims to investigate the effect of mental difficulties on the intensity of SNS use to bridge this gap.

The next section presents a review of the related work and the research model. Afterward, research methodology and data information are covered in section research methodology. Section research results shows the findings of the study. Moreover, Section discussion describes the discussion. Finally, section conclusion details the theoretical and practical implications of the research, followed by limitations and future research directions.

## Theoretical Background and Hypotheses Development

### Theoretical Background

Numerous studies have investigated the roles, impacts, and human behaviors of SNS since the COVID-19 outbreaks (Chang et al., 2020; Yoneoka et al., 2020; Zuo et al., 2020; Vall-Roqué et al., 2021). Zuo et al. (2020) verified that sharing physical activity experiences on SNS significantly affects social connectedness, positive self-presentation, and positive feedback during pandemics. It is also found that positive self-presentation significantly influences positive feedback. Vall-Roqué et al. (2021) examined the role of COVID-19 lockdown in deriving SNS use, low self-esteem, and body image disturbances. SNS use significantly influences body dissatisfaction, drive for thinness, and low self-esteem in the younger age group (14–24 years). Qin et al. (2020) argued that SNS enhances the relationship between people and society. Chang et al. (2020) clarified the factors that affect the COVID-19 pandemic compliance intention in the case of the citizens who have been quarantined or subjected to restricted mobility to prevent COVID-19. Active sharing of information through SNS during the pandemic was validated to enhance self-efficacy and perceived avoidability, resulting in positive thinking. Yoneoka et al. (2020) revealed a positive association between the number of COVID-19 cases and self-reported fevers of SNS users, implying that massive monitoring would help to capture the scale of the COVID-19 catastrophe.

Social distancing is the most representative and effective social measure for COVID-19 prevention. Several scholars have studied the attitudes, intentions, and behaviors toward social distancing. Hagger et al. (2020) proposed an extended social cognition model to examine the predictors of social distancing intention and behavior during COVID-19. They found that subjective norm, moral norm, and perceived behavioral control (PBC) are consistent predictors of social distancing intention. Adiyoso and Wilopo (2020) verified the significance of risk perception on social distancing attitudes in the context of COVID-19. They revealed that risk perception influences perceived behavioral control stronger in younger individuals than older people. Kawashima et al. (2021) examined the telework implementation and fever rate as a social distancing measure using the data gathered from SNS users. Company employees in the non-teleworker group showed statistically higher fever rates than the telework group.

Several works have shown that disaster preparedness and health behavior are determined by risk perception (Adiyoso and Kanegae, 2013; Bae and Chang, 2021; Savadori and Lauriola, 2021). A great deal of work on psychometrics has asserted that there are two fundamental procedures in which people perceive risk (Epstein, 1994; Sjöberg, 1998; Finucane et al., 2000; Slovic et al., 2004; Trumbo et al., 2016). Sjöberg (1998) noted that affective risk perception refers to an individual's anxiety about exposure to a particular risk. He also described cognitive risk perception as a person's perceived susceptibility to risks. Slovic et al. (2004) stated that risk as feelings represents a person's instinctive reactions to threat and risk as analysis is based on reason, logic, and deliberative processes. Affective risk perception is similar to risk as feeling and cognitive risk perception seems like risk as analysis. Risk perception has proven to have a positive correlation with behavioral intention (Floyd et al., 2004). Savadori and Lauriola (2021) investigated the relationship between risk perception and protective behaviors during the COVID-19 crisis. They uncovered that both feelings of risk and risk analysis are significantly associated with social distancing behavior. Bae and Chang (2021) validated the impact of COVID-19 risk perception on behavioral intention toward preventive tourism. They figured out that both affective risk perception and cognitive risk perception affect significant behavioral intention.

Cabin fever describes the stressful temper combined with inertia when a person experiences confinement over a long period (Fritscher, 2020a). People got restless, irritable, and lonely when they are in lack of social interaction and isolation (Hartwell-Walker, 2020). The COVID-19 pandemic might cause cabin fever syndrome because movement and socialization are being restricted. Several studies showed that the COVID-19 outbreak and its associated quarantine would be related to anxiety, depression, disturbed sleep, and post-traumatic stress disorder (Liang et al., 2020; Rajkumar, 2020). Estacio et al. (2020) validated the impacts of the implementation of community isolation on cabin fever syndrome. They found that majority of the participants experience manifestations of cabin fever. It was also observed that the female has difficulty in concentrating and sudden food cravings. Chakraborty et al. (2020) explored the psychological impact on SNS usage intensity by modifying cognitive dissonance theory. They developed the psychological impact as the second-order construct by combining cabin fever syndrome, loneliness, COVID-19 fear, and traumatic stress. The psychological impact was found to have significance on SNS usage intensity in the 21–35 years group and the students learning online group.

A number of research on SNS have explored the role of demographic variables such as gender, age, and income in explaining SNS behavior (Ji et al., 2014; Kim and Yoo, 2016; Vall-Roqué et al., 2021). Kim and Yoo (2016) examined the impacts of using SNS. The authors identified the effects of age and gender differences in those impacts. They found that there are significant differences along with age and gender in the effects of SNS usage. Ji et al. (2014) clarified social networking behaviors among younger and older adolescents regarding age, gender, and personality. They found that their latent utilization, socializing, and privacy disclosure SNS behaviors were influenced by age, gender, and personality. Asghar et al. (2022) analyzed social media tools for the development of pre-service health science researchers during COVID-19. They demonstrated that communication and multimedia significantly affect research completeness.

### Research Model and Hypotheses

**Figure 1:**
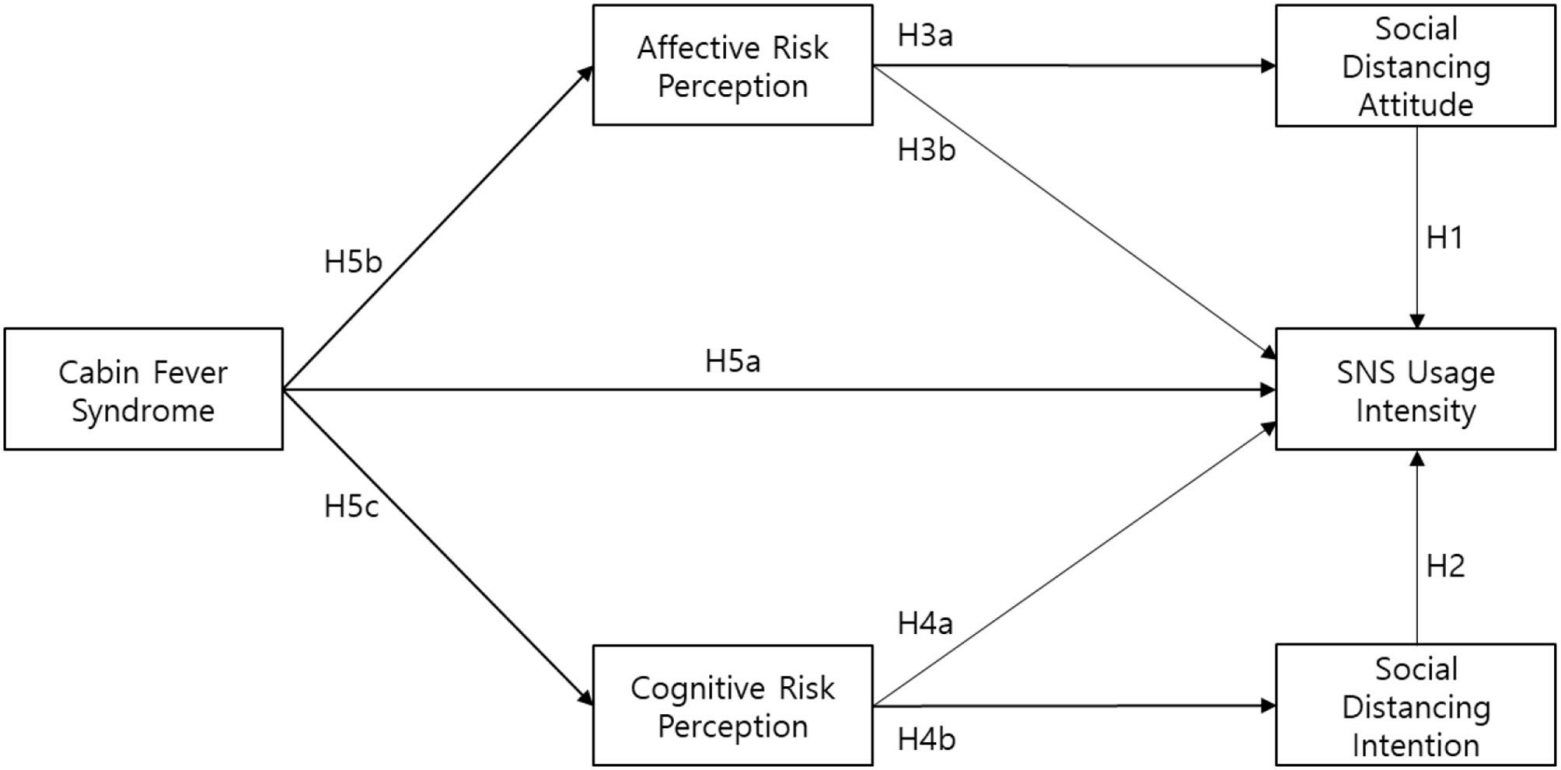
Research model.

Figure 1 depicts the theoretical framework for investigating the key factors of SNS usage intensity. This study posits that social distancing attitude, social distancing intention, affective risk perception, cognitive risk perception, and cabin fever syndrome as determinants that develop SNS usage intensity. As demographic factors have been employed in explaining user behaviors toward information system usage (Venkatesh et al., 2003) and SNS (Vall-Roqué et al., 2021), this article reflects gender and age as control variables in the conceptual model. The final research model is shown in Figure 1.

#### Social Distancing Attitude

Social distancing attitude represents the attitude people hold concerning complying with social distancing among people, as a means to inhibit COVID-19 infection (Williams et al., 2015; Fong et al., 2020). The stronger the attitude toward social distancing SNS users have, the more likely they would be to stay in the allowed space compared to those who do not. Since SNS can be accessed easily with a computer or smartphone, it can be used easily even at home or in an isolated location. The greater the degree of attitude toward social distancing, the more likely they might be to use SNS more actively. Therefore, one can expect that social distancing attitude has an impact on SNS usage intensity.

Hypothesis H1. Social distancing attitude significantly influences SNS usage intensity.

#### Social Distancing Intention

Social distancing intention refers to the degree to which people want to practice social distancing to prevent COVID-19 infection (Williams et al., 2015; Fong et al., 2020). CAVSD (Cognitive appraisal of voluntary social distancing compliance), which consists of social distancing attitude and social distancing intention, serves as the key factor of SNS usage intensity in the age group under 21 years (Chakraborty et al., 2020). In some cases, attitude and intention for social distancing are not mutually significant (Adiyoso and Wilopo, 2020). Therefore, it is necessary to identify the roles of social distancing attitude and social distancing intention separately. The greater the intention of social distancing citizens hold, the more likely they are to avoid going out or contacting others. Since SNS is a representative channel that enables communication and social exchange with others even when isolated, the people who have more intention of social distancing would raise the degree of SNS usage intensity. Thus, social distancing intention is expected to be a dominant factor in elevating the level of SNS usage intensity.

Hypothesis H2. Social distancing intention significantly influences SNS usage intensity.

#### Affective Risk Perception

Affective risk perception deals with the mood experienced when thinking about a hazard (Ferrer et al., 2018; Kaufman et al., 2019). It is the most powerful driver of protection motivation across a variety of hazards (Janssen et al., 2014). It has been found to determine the attitude toward preventive behavior in the context of COVID-19 (Bae and Chang, 2021). Feelings of risk, similar to affective risk perception, have a positive impact on social distancing (Savadori and Lauriola, 2021). SNS users with higher levels of affective perceptions of risk are likely to try to hinder infection. Thus, they judge that social distancing is effective. They would also refrain from going out and avoid contact with others to participate in preventive measures. This leads to an increase in the use of SNS, a communication channel in the isolated space. Therefore, affective risk perception is believed to positively affect both SNS usage intensity and social distancing attitude.

Hypothesis H3a. Affective risk perception significantly influences SNS usage intensity.

Hypothesis H3b. Affective risk perception significantly influences social distancing attitude.

#### Cognitive Risk Perception

Cognitive risk perception refers to the degree of risk when a person thinks about a particular disaster or hazard (Trumbo et al., 2016). It has a significant impact on behavioral intention to hamper COVID-19 (Bae and Chang, 2021). Risk analysis, resembling cognitive risk perception, significantly affects social distancing behavior (Savadori and Lauriola, 2021). The intention to get vaccinated against diseases is stronger among people perceiving the probability of getting infected as higher (Brewer et al., 2007). As a consequence, recognizing the probability of a higher risk of COVID-19, SNS users would take part in social distancing more actively. They might also try to stay in isolated spaces, which will increase the frequency of SNS activities. Thus, it is expected that cognitive risk perception affects both SNS usage intensity and social distancing intention.

Hypothesis H4a. Cognitive risk perception significantly influences SNS usage intensity.

Hypothesis H4b. Cognitive risk perception significantly influences social distancing intention.

#### Cabin Fever Syndrome

Cabin fever syndrome is justified as a negative temper combined with claustrophobic lethargy when a person is caught in a quarantined space for a long period (Fritscher, 2020b; Robinson, 2020). In the context of COVID-19, there is no place to stay long except at home or work. People would feel frustrated and uncomfortable when they can not get out of a particular place for a long time (Hartwell-Walker, 2020; Rajkumar, 2020). The SNS users with greater cabin fever syndrome had higher perceptions of risk for COVID-19 because they would think that isolation is required due to COVID-19. In addition, the stronger the cabin fever syndrome, the harder they might communicate on SNS to relieve the feeling of isolation and closure they experience. Thus, this study expects that cabin fever syndrome plays a key role in forming SNS usage intensity, affective risk perception, and cognitive risk perception.

Hypothesis H5a. Cabin fever syndrome significantly influences SNS usage intensity.

Hypothesis H5b. Cabin fever syndrome significantly influences affective risk perception.

Hypothesis H5c. Cabin fever syndrome significantly influences cognitive risk perception.

## Research Methodology

### Instrument Development

All indicators corresponding to each factor within the research framework were selected from previously validated measures. The measurement items were modified to fit the case of SNS. Before the main survey was implemented, experts in the field of information systems and social science reviewed the questionnaire to assure logical order, wording, and question ambiguity. A pilot survey was performed to confirm the validity and reliability of the measures and to confirm their completeness. A total of 20 University students participated in the pilot test (Julious, 2005). The feedback was used to correct some indicators to ensure they were comprehensible to all the respondents. Each item was measured with a 7-point Likert scale, ranging between 1 (“strongly disagree”) and 7 (“strongly agree”). Table A lists the survey items.

#### SNS Usage Intensity

The four statements related to the SNS usage intensity were adapted from Eid and Al-Jabri (2016). The examples of these items included “During social distancing/lockdown I am using social networking (SN) more than normal.” and “During social distancing/lockdown I am logging into my SN sites more frequently.”

#### Social Distancing Attitude

The three statements related to the social distancing attitude were adapted from Williams et al. (2015). The examples of these items included “In my opinion, the use of social distancing will have a positive impact to control COVID 19.” and “The use of social distancing is beneficial for the care of the patients.”

#### Social Distancing Intention

The three statements related to the social distancing intention were adapted from Williams et al. (2015). The examples of these items included “I have the intention to use social distancing when it becomes useful to avoid COVID 19.” and “I have the intention to use social distancing when necessary to provide good results to avoid COVID-19.”

#### Affective Risk Perception

The four statements related to the affective risk perception were adapted from Bae and Chang (2021) and Brug et al. (2004). The examples of four items included “I am worried that I will contract COVID-19.” and “I am worried about my family members contracting COVID-19.”

#### Cognitive Risk Perception

The four statements related to the cognitive risk perception were adapted from Bae and Chang (2021) and Brug et al. (2004). The examples of four items included “There is a high likelihood of acquiring COVID-19 in general.” and “There is a high likelihood that I will acquire COVID 19 compared to other people.”

#### Cabin Fever Syndrome

The four statements related to the social distancing intention were adapted from Fritscher (2020b) and Robinson (2020). The examples of four items included “I feel restless staying at home.” and “I have trouble concentrating while staying at home during social distancing/lockdown.”

### Data Collection

The theoretical model was empirically verified by the use of data collected from the online survey. A survey was conducted for University students who had been using SNS during the COVID-19 pandemic. The participants were selected using a convenience sampling technique that has the advantage of being easily applied (Rasool et al., 2020). Students from four universities in Da Nang, Vietnam, received an online link to the survey. Vietnam was implementing social distancing during this period and University education was conducted entirely online (Vietnam Briefing, 2022). Therefore, it could be possible to accurately measure the risk perception and cabin fever syndrome of University students. The online questionnaire was distributed and collected from 16 September to 22 September 2021. Several professors helped collect data in their class. The purpose and aim of this study were explained on the first page of the questionnaire. This research informed that the collected data would be used only for academic purposes. Following the Statistics Act, participants were informed that their personal information of them would not be disclosed. The informed consent was specified on the same page. Only the consenting students voluntarily participated in the survey. Informants were provided an appropriate environment and time to think about fully the contents of the questionnaire and respond clearly. After discarding incomplete and aberrant responses, 271 responses were used for analysis. This research employed an a-priori sample size calculator to confirm the minimum requirement for structural equation models (DanielSoper.com)^1^. Inputting the required information such as 0.1 anticipated effect size, 80% desired statistical power level, 6 number of latent variables, 22 number of observed variables, as well as 0.05 probability level, the minimum required sample size is 123. Since the sample size of this study is 271, this requirement is met as well. Among the final samples, 116 (42.8%) responses were male and 155 (57.2%) responses were female. The mean age of the final sample was 21.78 years with a standard deviation of 2.67. The demographic information of the final data is described in Table 1.

**Table 1:** Sample characteristics.

**Demographics**	**Item**	**Subjects (*N* = 271)**	
		**Frequency**	**Percentage**
Gender	Male	116	42.8
	Female	155	57.2
Age	19 or younger	53	19.6
	20–23	171	63.1
	24 or older	47	17.3

## Research Results

The data analysis was conducted using structural equation modeling (SEM). In SEM, there are two techniques, which are a variance-based technique and a covariance-based technique. This research used the partial least squares (PLS) method, a variance-based technique, because the research model has not been demonstrated in the literature (Hair et al., 2011). The PLS provides the advantage of suggesting fewer restrictions on the sample size and residuals compared to covariance-based techniques (Chin, 1998; Hair et al., 2012). The PLS has been extensively selected as a tool in the IS field (Chin et al., 2003). This study carried out a two-step analysis to test the measurement model and the structural model by using SmartPLS 3.3.9 (Ringle et al., 2014).

### Common Method Bias

This research used the principal axis factoring method with Harman's one-factor test, ensuring that none of the factors individually explains the majority of the variance (Podsakoff, 2003). The results showed that the first factor explains 26.3% of the variance. No significant common method bias was found.

### Measurement Model

To test a measurement model, this study analyzes the reliability, convergent validity, and discriminant validity of the measurements. To evaluate reliability, Cronbach's alpha and composite reliability (CR) were calculated. As described in Table 2, Cronbach's alpha and CR of all the constructs exceeded the recommended threshold of 0.7 (Nunnally, 1978).

**Table 2:** Scale reliabilities.

**Construct**	**Items**	**Mean**	**St. Dev**.	**Factor loading**	**Cronbach's Alpha**	**CR**	**AVE**
SNS usage intensity	SUI1	5.019	1.829	0.935	0.871	0.912	0.724
	SUI2	5.058	1.880	0.941			
	SUI3	4.519	1.834	0.794			
	SUI4	4.798	1.649	0.711			
Social distancing attitude	SDA1	5.490	1.352	0.818	0.778	0.869	0.689
	SDA2	5.077	1.504	0.846			
	SDA3	4.663	1.627	0.827			
Social distancing intention	SDI1	6.106	0.950	0.966	0.952	0.969	0.912
	SDI2	6.096	0.946	0.945			
	SDI3	6.192	0.921	0.953			
Affective risk perception	ARP1	4.663	1.864	0.918	0.910	0.936	0.786
	ARP2	5.423	1.591	0.869			
	ARP3	4.760	1.707	0.878			
	ARP4	5.212	1.479	0.880			
Cognitive risk perception	CRP1	4.452	1.709	0.865	0.784	0.858	0.604
	CRP2	3.231	1.564	0.738			
	CRP3	4.462	1.748	0.739			
	CRP4	3.721	1.638	0.759			
Cabin fever syndrome	CFS1	3.125	1.752	0.865	0.703	0.778	0.473
	CFS2	3.990	2.021	0.635			
	CFS3	3.885	2.006	0.633			
	CFS4	3.462	1.911	0.581			

Convergent validity was ensured by investigating both the average variance extracted (AVE) and the factor loadings of the items related to each construct. The AVE must be over 0.5, meaning that the latent variables account for more than half of the variance of their items (Henseler et al., 2009; Hair Jr et al., 2014). AVE values of all constructs, except cabin fever syndrome, were deemed to exhibit adequate, with a validity above the expected threshold. This study retains cabin fever syndrome since other estimates such as Cronbach's alpha and CR were well over the threshold (0.703 and 0.778, respectively). The factor loadings ranged from 0.581 to 0.966.

Finally, the discriminant validity was confirmed through the Fornell and Larcker (1981) and the heterotrait-monotrait ratio of correlations (HTMT) (Henseler et al., 2015). All the AVE values are higher than the correlation value for that column or row, ensuring the presence of discriminant validity (Fornell and Larcker, 1981). Table 3 shows the correlation matrix and the results of the Fornell and Larker evaluation.

**Table 3:** Correlation matrix and discriminant assessment.

**Constructs**	**1**	**2**	**3**	**4**	**5**	**6**
1. SNS usage intensity	0.851					
2. Social distance attitude	0.221	0.830				
3. Social distance intention	0.080	0.581	0.955			
4. Affective risk perception	0.104	0.383	0.332	0.887		
5. Cognitive risk perception	0.110	0.271	0.203	0.739	0.777	
6. Cabin fever syndrome	0.243	0.059	−0.063	0.358	0.396	0.688

*Diagonal values are the square root of AVE*.

The HTMT values for all factors were below the threshold of 0.95 (Ab Hamid et al., 2017) as depicted in Table 4.

**Table 4:** HTMT.

**Constructs**	**1**	**2**	**3**	**4**	**5**	**6**
1. SNS usage intensity						
2. Social distance attitude	0.264					
3. Social distance intention	0.125	0.685				
4. Affective risk perception	0.161	0.444	0.363			
5. Cognitive risk perception	0.152	0.350	0.214	0.865		
6. Cabin fever syndrome	0.315	0.229	0.169	0.294	0.386	

This study assessed the predictive relevance *Q*^2^ by using Blindfolding in SmartPLS. The omission distance D was set as 7. Table 5 describes the results. All values of *Q*^2^ were larger than the cut-off of 0. The cross-validated redundancy measures the capability of the path model to predict the endogenous measuring items indirectly from the prediction of their latent variables using the related structural relations. It is only computed for the endogenous variables.

**Table 5:** Results of redundancy analysis.

**Constructs**	**Cross-validated redundancy**	
SNS usage intensity	0.117	Moderate predictive power
Social distancing attitude	0.096	Moderate predictive power
Social distancing intention	0.027	Moderate predictive power
Affective risk perception	0.089	Moderate predictive power
Cognitive risk perception	0.070	Moderate predictive power
Cabin fever syndrome		

### Structural Model and Hypothesis Testing

An SEM was carried out to assess the proposed relationships among constructs through PLS. A bootstrap resampling technique (5,000 subsamples) was used to validate the significance of the hypotheses within the theoretical framework.

SEM did not show multicollinearity issues as the variance information factor (VIF) values were below 5. The VIF values for constructs were Social distancing attitude = 1.65, Social distancing intention = 1.66, Affective risk perception = 2.60, Cognitive risk perception = 2.34, and Cabin fever syndrome = 1.25.

**Figure 2:**
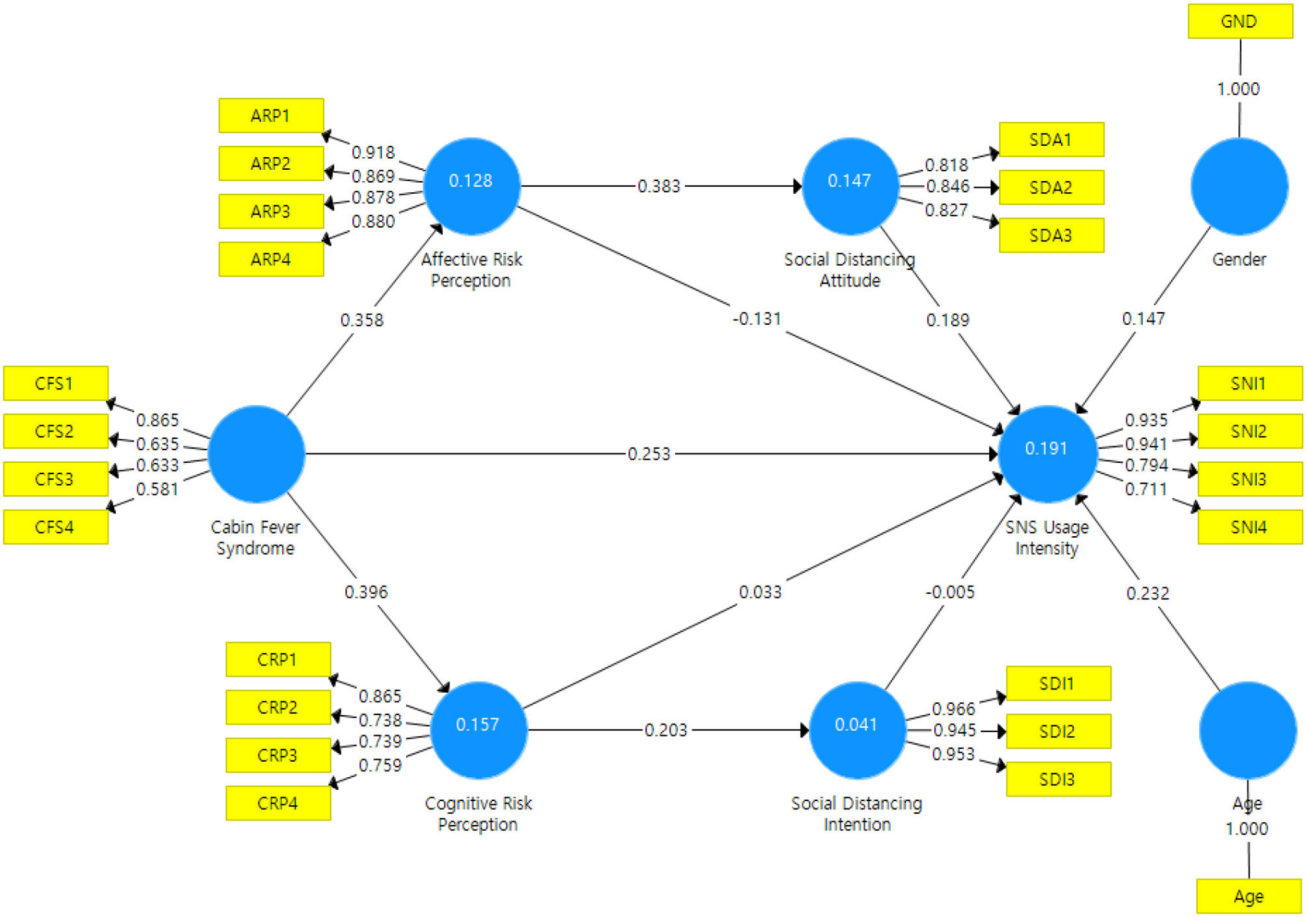
Results of structural model.

Figure 2 shows the main path coefficients and explained endogenous variables' variances (*R*^2^) for the structural model. Contrary to predictions, social distancing does not affect social network intensity, failing to support H1. Social distancing intention is not significantly related to social network intensity, failing to accept H2. Affective risk perception does not influence social network intensity, while it significantly affects social distancing attitude. Thus, H3a is not accepted and H3b is supported. Cognitive risk perception is not significantly related to social network intensity, whereas has a significant impact on social network intensity. Therefore, H4a is not supported and H4b is accepted. Cabin fever syndrome has a significant effect on social network intensity, affective risk perception, and cognitive risk perception, thereby supporting H5a, H5b, and H5c. Overall, the research model accounted for ~19% (0.191) of the variance in social network intensity, 14.7% (0.147) of the variance in social distancing attitude, and 4.1% (0.041) of the variance in social distancing intention.

The estimated coefficient of determination in this study is relatively low. This may be due to the following reasons. First, the present work exploratorily verified the paths that have not been attempted in previous studies. *R*^2^ may be low because the hypothesis was newly established and analyzed. Second, this research considered only University students as the study subjects. The University student group may use SNS more frequently than other groups. This particularity may have undermined the generality of the model. Table 6 summarizes the results of hypothesis testing.

**Table 6:** Results of hypothesis testing.

**H**	**Cause**	**Effect**	**Coefficient**	***t***	**Hypothesis**
H1	Social distancing attitude	SNS usage intensity	0.189	0.239	Not Supported
H2	Social distancing intention	SNS usage intensity	−0.005	0.967	Not Supported
H3a	Affective risk perception	SNS usage intensity	−0.131	0.464	Not Supported
H3b	Affective risk perception	Social distancing attitude	0.383	0.000	Supported
H4a	Cognitive risk perception	SNS usage intensity	0.033	0.819	Not Supported
H4b	Cognitive risk perception	Social distancing intention	0.203	0.027	Supported
H5a	Cabin fever syndrome	SNS usage intensity	0.358	0.001	Supported
H5b	Cabin fever syndrome	Affective risk perception	0.396	0.000	Supported
H5c	Cabin fever syndrome	Cognitive risk perception	0.253	0.047	Supported

Moreover, gender has no significant effects on SNS usage intensity. Age is significantly associated with SNS usage intensity. The plausible reason for this phenomenon is that older students have fewer compulsory classes than the younger group and thereby have more time to use SNS long. Besides, higher grades might use SNS more to share information and interact with acquaintances who are graduating or engaged in social activities.

*F*^2^ depicts the contribution of each construct among the relationships within the research model (Bhutta et al., 2019). It also considers the significance of one construct on another construct along with the degree of its effectiveness. The score of the *F*^2^ should be <0.02 to hold a significant relationship. Table 7 describes the *F*^2^ scores. In some effects, the criteria are not met. Nevertheless, the next verification was conducted since this study intended an exploratory analysis to develop a new research model.

**Table 7:** Results of *F*^2^.

	**1**	**2**	**3**	**4**	**5**	**6**
1. SUI						
2. SDA	0.027					
3. SDI	0.000					
4. ARP	0.008	0.172				
5. CRP	0.001		0.043			
6. CFS	0.063			0.147	0.186	

This research tested the model fit indices of the structural model. Indices were standardized root mean square residual (SRMR), RMS_theta, Normative fit index (NFI), and Goodness-of-Fit (GoF). SRMR should be <0.08 and RMS_theta should be <0.1 (Hair et al., 2019). NFI must be over 0.95 (Hu and Bentler, 1999 cutoff). SRMR was 0.088 and RMS_theta was 0.180. NFI was 0.701. GoF is defined as “how well the specified model reproduces the observed covariance matrix among the indicator items” (Hair et al., 1998). The model's GoF for this study was 0.305, indicating a medium level of fit (Wetzels et al., 2009). SRMR, RMS_theta, and NFI do not present a satisfactory level of criteria. Nonetheless, the results of hypothesis testing are discussed because the research model is not developed based on the existing theories.

## Discussion

The current study aimed to identify the effects of cabin fever syndrome and risk perception on SNS usage intensity through social distancing. This has been completed by developing the conceptual framework and validating it for University students.

The results showed that social distancing attitude and social distancing intention are not significantly related to SNS usage intensity. Social media offers a substitute for socialization in virtual spaces for face-to-face socialization (Kujath, 2011). Particularly, students actively socialized themselves on social media with their friends before the COVID-19 pandemic (Gao et al., 2020). The use of social media such as SNS may increase because face-to-face relationships are reduced under social distancing conditions. Chakraborty et al. (2020) revealed that CAVSD positively affects SNS usage intensity only in the age group of under 20 years. People under 20 years are subject to compulsory education. They would spend more time at home than older groups and might increase the use of social networking. On the other hand, those over the age of 20 years have other social life. Even if social distancing is enforced, they can operate in spaces other than home. They have more alternatives to act or spend time than those who are under 20 years. Therefore, social network usage intensity would not change more than those under 20 years.

The findings of the research revealed that SNS usage intensity is not influenced by affective risk perception and cognitive risk perception. Previous studies support that people might have a relatively accurate risk perception during COVID-19 (Bodrud-Doza et al., 2020; Kuang et al., 2020). Chakraborty et al. (2020) proved that psychological impact significantly enhances the level of SNS usage intensity in people in their early 20s. University students who suffer from frustration or loneliness due to COVID-19 would solve their mental difficulties by increasing the use of SNS. However, students who perceive the risk as greater could be lowering their risk perception by performing health protection activities, not SNS.

The results of the present study pointed out that affective risk perception and cognitive risk perception have a positive impact on social distancing attitudes. It was also validated that risk perception is the salient determinant of social distancing attitude (Adiyoso and Wilopo, 2020). Similarly, the significant association between risk perception and preventive health behavior was also supported in previous research (Dryhurst et al., 2020; Savadori and Lauriola, 2021). A possible explanation is the fact that the stronger students feel emotionally at risk for COVID-19, the higher the level of their attitude toward social distancing. Moreover, University students with a higher level of cognitive risk perception of COVID-19 may increase their intention toward social distancing. SNS activity does not directly reduce risk. Meanwhile, social distancing is a health protection behavior that decreases the probability of droplet infection. Students would engage in health-protective actions when affective risk perception and cognitive risk perception are raised. Previous research on the roles of affective risk perception and cognitive risk perception on health behavior has yielded mixed results (Bae and Chang, 2021; Savadori and Lauriola, 2021). The difference between the results of this work and former studies could be due to the type of health protection behaviors such as social distancing, contactless tours, and hygiene.

In addition, it was supported that cabin fever syndrome affected affective risk perception, cognitive risk perception, and SNS usage intensity. These results could be accredited to the following reasons. Students who feel uncomfortable in an isolated space are more intensive in SNS activities. They may use cyberspace and channels to communicate with friends and acquaintances to relieve isolation or stress. The significant correlation between psychological impact and SNS usage intensity was also supported in the former research (Chakraborty et al., 2020). Cabin fever syndrome also raised the level of risk perception for COVID-19. This indicates that the more lethargic or socially separated people feel in a confined place, the stronger they perceive the risk of COVID-19. Support from family and friends can enhance social media use and strengthen psychological resilience (Asghar et al., 2021). Combining this study with Asghar et al. (2021), one can find that cabin fever syndrome increases SNS use and social media use promotes psychological resilience. Therefore, it may be meaningful to design an appropriate social media environment and provide it to users to stabilize their mental status.

## Conclusion

### Implications for Researchers and Practitioners

This study offers several implications for researchers and practitioners. First, the present study examines the roles of social distancing attitude and social distancing intention to explain SNS usage intensity during COVID-19. Existing studies have identified factors influencing social distancing practices (Adiyoso and Wilopo, 2020; Hagger et al., 2020), observations of negative emotion (Xiao et al., 2020), and the moderating effect on e-learning (Saxena et al., 2021). The results of the study reveal that both social distancing attitude and social distancing intention are not significantly related to SNS usage intensity in the 20s age group. This paper makes an academic contribution by revealing that the perception and behavior of social distancing among University students do not empirically affect the intensity of SNS use. Researchers can analyze groups such as office workers and freelancers to compare them with the results of this study. New results may be obtained by additionally illuminating groups that have changed working hours or conditions before and after the COVID-19 outbreak. SNS providers may be able to lower their priorities for factors related to social distancing when marketing to University students.

Second, this research newly contributed to academia by clarifying the impacts of affective risk perception and cognitive risk perception in shaping SNS usage intensity, social distancing attitude, and social distancing intention. It was found that risk perception affects social distancing. However, risk perception was shown to not affect the intensity of SNS use. In line with the results of this research, previous research has verified the significant effect of risk perception on social distancing (Adiyoso and Wilopo, 2020; Xie et al., 2020; Savadori and Lauriola, 2021). It will be meaningful for scholars to divide and analyze factors that influence health protection behavior and the frequency of SNS use, respectively. If future research identifies the role of social norms and regulatory environments as a leading factor of social distancing along with risk perception, it may be possible to derive valuable implications for the public interest. At the same time, researchers can obtain beneficial results for the growth of the SNS market if they prove the effects of peer influence and social content as a deciding factor in SNS usage intensity. People may participate in social distancing if they become aware of the danger. Therefore, the disaster management headquarter should continue to inform citizens of the lethality of COVID-19 and the deadly nature of the disease. The disaster control tower needs to disclose the number of confirmed cases, infection routes, and new variants regarding the spread of COVID-19. By constantly updating the damage caused by COVID-19, it will be possible to form people's risk perception and realize social distancing.

Finally, this paper contributes to the literature by investigating the impact of cabin fever syndrome on SNS usage intensity, affective risk perception, and cognitive risk perception. The results show that cabin fever syndrome significantly determines SNS usage intensity, affective risk perception, and cognitive risk perception. It was found that the support of family and friends improves the level of psychological resilience via social media (Asghar et al., 2021). Therefore, it would be useful if policymakers provide an appropriate social media environment for people's mental recovery. They can examine the degree of claustrophobic restlessness of citizens and resolve their sense of helplessness. In addition, SNS providers need to launch new services or games that can alleviate these emotional troubles by conducting events such as small surveys that can identify users' levels of cabin fever syndrome.

### Limitations and Future Research

Some limitations need to be claimed in this study. First, the analysis was performed only on the 20s group of SNS users. In future research, it is necessary to verify the reliability and validity of the research model by conducting a questionnaire for all age groups to provide a more comprehensive understanding. Second, the intensity of users might vary according to the purpose of use. There are many types of purposes such as pleasure, personal use, a commercial channel, and utilitarian needs. In particular, groups that use SNS commercially may have increased the frequency of use due to the increase in the non-face-to-face work environment after COVID-19. Therefore, it is needed to test the hypothesis according to the user's purpose of use. Finally, this study considered social distancing, risk perception, and cabin fever syndrome caused by COVID-19 to explain the intensity of SNS. In future studies, integrating the unique characteristics of SNS that have been changed due to COVID-19 would also enhance the explanatory power of the model.

## Author's Note

HJ received his B.S., M.S., and Ph.D. degrees from the Korea Advanced Institute of Science and Technology (KAIST) in 2004, 2006, and 2012, respectively. He was an assistant professor at the College of Business Administration, Dong-A University, South Korea during 201396-2018. His current affiliation is RealSecu which provides IT network security services. His research interests are 4.0 industry, smart lighting, IT security, collaborative filtering, web data analysis, and Internet information. He has published in Journal of the Knowledge Economy, Journal of Business and Industrial Marketing, Technology Analysis and Strategic Management, International Journal of Human-Computer Interaction, among others.

## Data Availability Statement

The original contributions presented in the study are included in the article/supplementary material, further inquiries can be directed to the corresponding authors.

## Author Contributions

The author confirms being the sole contributor of this work and has approved it for publication.

## Conflict of Interest

HJ was employed by RealSecu.

## Publisher's Note

All claims expressed in this article are solely those of the authors and do not necessarily represent those of their affiliated organizations, or those of the publisher, the editors and the reviewers. Any product that may be evaluated in this article, or claim that may be made by its manufacturer, is not guaranteed or endorsed by the publisher.

## Footnotes

^1^DanielSoper.com. Free Statistics Calculators Available at https://www.danielsoper.com/statcalc/default.aspx (accessed on 8 December 2021).

## Appendix

**Table A:** Survey instrument.

**Construct**	**Item**	**Mean**
SNS Usage Intensity Eid and Al-Jabri, 2016	SUI1	During social distancing/lockdown, I am using social networking (SN) more than normal.
	SUI2	During social distancing/lockdown, I am logging into my SN sites more frequently.
	SUI3	During social distancing/lockdown, I am sending/forwarding messages to my friends more than normal.
	SUI4	During social distancing/lockdown, I am using social networking sites for reading news and attending social gathering.
Social Distancing Attitude Williams et al., 2015	SDA1	In my opinion, the use of social distancing will have a positive impact to control COVID-19.
	SDA2	The use of social distancing is beneficial for the care of the patients.
	SDA3	I find it interesting to use social distancing for the control of COVID-19.
Social Distancing Intention Williams et al., 2015	SDI1	I have the intention to use social distancing when it becomes useful to avoid COVID-19
	SDI2	I have the intention to use social distancing when necessary to provide good results to avoid COVID -19
	SDI3	I have the intention to use social distancing for the care of myself and others
Affective Risk perception Brug et al., 2004; Bae and Chang, 2021	ARP1	I am worried that I will contract COVID-19.
	ARP2	I am worried about my family members contracting COVID-19.
	ARP3	I am worried about COVID-19 occurring in my region.
	ARP4	I am worried about COVID-19 emerging as a health issue.
Cognitive Risk Perception Brug et al., 2004; Bae and Chang, 2021	CRP1	There is a high likelihood of acquiring COVID-19 in general.
	CRP2	There is a high likelihood that I will acquire COVID-19 compared to other people.
	CRP3	There is a high likelihood of acquiring COVID-19 compared to other diseases.
	CRP4	There is a high likelihood of dying from COVID-19.
Cabin Fever Syndrome Fritscher, 2020b; Robinson, 2020	CFS1	I feel restless staying at home.
	CFS2	I have trouble concentrating while staying at home during social distancing/lockdown.
	CFS3	I have food cravings while staying at home during social distancing/lockdown.
	CFS4	I have a feeling of social isolation while staying at home during social distancing/lockdown.
